# Plasma Amino Acid Profiling Identifies Specific Amino Acid Associations with Cardiovascular Function in Patients with Systolic Heart Failure

**DOI:** 10.1371/journal.pone.0117325

**Published:** 2015-02-06

**Authors:** Daihiko Hakuno, Yasuhito Hamba, Takumi Toya, Takeshi Adachi

**Affiliations:** 1 Division of Cardiology, Department of Internal Medicine, National Defense Medical College, Tokorozawa, Saitama, Japan; 2 Department of Laboratory Medicine, National Defense Medical College, Tokorozawa, Saitama, Japan; Merck & Co., UNITED STATES

## Abstract

**Background:**

The heart has close interactions with other organs’ functions and concomitant systemic factors such as oxidative stress, nitric oxide (NO), inflammation, and nutrition in systolic heart failure (HF). Recently, plasma amino acid (AA) profiling as a systemic metabolic indicator has attracted considerable attention in predicting the future risk of human cardiometabolic diseases, but it has been scarcely studied in HF.

**Methods:**

Thirty-eight stable but greater than New York Heart Association class II symptomatic patients with left ventricular (LV) ejection fraction <45% and 33 asymptomatic individuals with normal B-type natriuretic peptide (BNP) value were registered as the HF and control groups, respectively. We analyzed fasting plasma concentrations of 41 AAs using high-performance liquid chromatography, serum NO metabolite concentration, hydroperoxide and high-sensitivity C-reactive protein measurements, echocardiography, and flow-mediated dilatation.

**Results:**

We found that 17 AAs and two ratios significantly changed in the HF group compared with those in the control group (p < 0.05). In the HF group, subsequent univariate and stepwise multivariate analyses with clinical variables revealed that Fischer ratio and five specific AAs, ie, monoethanolamine, methionine, tyrosine, 1-methylhistidine, and histidine have significant correlation with BNP, LV ejection fraction, LV end-diastolic volume index, inferior vena cava diameter, the ratio of early diastolic velocity of the mitral inflow to mitral annulus, and BNP, respectively (p < 0.05). Interestingly, further exploratory factor analysis categorized these AAs into hepatic-related (monoethanolamine, tyrosine, and Fischer ratio) and skeletal muscle-related (histidine, methionine, and 1-methylhistidine) components. Some categorized AAs showed unique correlations with concomitant factors: monoethanolamine, tyrosine, and Fischer ratio with serum NO concentration; histidine with serum albumin; and 1-methylhistidine with flow-mediated dilatation (p < 0.05).

**Conclusions:**

Plasma AA profiling identified correlations of specific AAs with cardiac function and concomitant factors, highlighting the cardio-hepatic-skeletal muscle axis in patients with systolic HF.

## Introduction

Heart failure (HF) is a growing burden in terms of public health and medical costs and affects 2.4% of the adult population in the United States [1] and presumably 1% in the near future in Japan [2]. The functions of the heart and other organs such as kidneys, liver, skeletal muscle, fat, and blood vessels are often correlated with each other, suggesting possible mechanistic interactions in the pathogenesis in systolic HF syndrome [3–6]. For example, in hepatic function, a decrease in serum albumin concentration and an increase in transaminases, biliary enzymes, and the Model for End-Stage Liver Disease (MELD) score are significantly associated with mortality and the requirement for ventricular assist devices or heart transplantation in HF [7–11]. HF also complicates skeletal muscle wasting and abnormal muscle metabolism because of biochemical and bioenergetic alterations, leading to exercise intolerance and worse prognosis [12–14].

Concomitantly, measures of systemic factors such as oxidative stress, nitric oxide (NO), inflammation, and nutrition are closely correlated with cardiac function and may modify or predict the prognosis of HF [15, 16].

Recently, the profiling of plasma metabolites including amino acids (AAs) by high-resolution mass spectroscopy or nuclear magnetic resonance spectroscopy has stimulated new research interest in cardiovascular diseases because such profiling allows researchers to explore novel biomarkers for future risk or prognosis, pathogenesis, and the identification of possible new therapeutic targets [17]. Plasma AA profiles are regarded as systemic metabolic indicator, whereas AA dynamics have distinguishing characteristics. More than half of the AA pool exists intracellularly in the skeletal muscle [18], and the metabolism of specific AAs seems to be organ specific: branched-chain AAs (BCAAs) in the skeletal muscle (>50%), brain, and adipose tissue [19] and aromatic AAs in the liver [20]. Researchers have made rigorous associations, e.g., between plasma AA profiling and the likelihood of future risk of cardiometabolic diseases, such as diabetes [21, 22] and coronary artery disease [23, 24]. In contrast, AA profiling in HF has scarcely been studied to date [25, 26].

Therefore, the present study was aimed to investigate plasma AA profiles and their correlations with cardiac function and concomitant systemic factors in patients with systolic HF.

## Methods

### Study design

This case–control study was performed in our university hospital and affiliated Tokorozawa Daiichi Hospital. The participants were 20–80 years old, and their estimated glomerular filtration ratio (eGFR) was >30 mL/min/1.73 m^2^. The eligibility criteria of the HF group included stable inpatients with symptoms greater than New York Heart Association class II, B-type natriuretic peptide (BNP) levels >40 pg/mL, and left ventricular ejection fraction (LVEF) <45% measured using modified Simpson’s method of echocardiography, regardless of whether patients were receiving an angiotensin-converting enzyme inhibitor, angiotensin II type I receptor blocker, or β-blocker. Unstable patients receiving intravenous inotropes or diuretics, nitrates, AAs, an albumin preparation, or blood transfusion were excluded. The eligibility criteria of the control group included asymptomatic individuals who received an annual medical check-up or asymptomatic outpatients whose BNP levels were <40 pg/mL. The subjects with pregnancy, inflammatory and autoimmune disease, active cancer, or co-medications with neprilysin inhibitors, glucocorticoids, sex steroids, antineoplastics, and β-adrenergic agonists were excluded from the study. Participants were recruited and data were collected during 1 year, and 38 patients with HF and 33 control subjects were enrolled.

This study complied with the Declaration of Helsinki. The study protocol was approved by the Institutional Review Board of National Defense Medical College Hospital (approval number 828), and written informed consent was obtained from all participants.

### Parameter Measurements

All participants fasted for at least 12 h before blood collection. For measurement of plasma concentrations of 41 AAs, blood samples were immediately separated to plasma by EDTA and kept frozen at −80°C. Analysis was performed by high-performance liquid chromatography with UV detector (ACQUITY UPLC, Waters Corp., Milford, MA, USA). We measured plasma BNP and serum concentrations of NO metabolites as NO_2_^−^ + NO_3_^−^, hydroperoxide, and high-sensitivity C-reactive protein (hsCRP). Plasma BNP and serum concentration of NO metabolites were analyzed by chemiluminescent enzyme immunoassay (PATHFAST BNP, LSI Medience Corp., Tokyo, Japan) and the NO_2_/NO_3_ Assay Kit-FX (Fluorometric, DOJINDO, Kumamoto, Japan), respectively. Hemoglobin and proteins were removed using a membrane filter (Amicon Ultra 10 kDa Ultracel, Millipore, Billerica, MA, USA) before initiation of the assay to prepare sample solutions. Then, the concentration was determined by the fluorometric method using NO_3_^−^ reductase and 2,3-diaminonaphthalene, according to the manufacturer’s instructions. The plasma concentration of hydroperoxide products was measured by the d-ROMs test (FREE carpe diem, Diacron International, Grosseto, Italy) according to the manufacturer’s instructions. A modified version of the MELD (MELD-XI) score [27] and geriatric nutritional risk index (GNRI) [28] were calculated as follows, respectively: 5.11 × (ln total bilirubin) + 11.76 × (ln creatinine) + 9.44, 14.89 × serum albumin + 41.7 × body mass index/22.

For the HF group, we examined flow-mediated dilatation by UNEXEF 38G (UNEX Corporation, Nagoya, Japan) and brachial-ankle pulse wave velocity as indicators of vascular endothelial function. Furthermore, we measured echocardiographic parameters of diameters and thickness of LV, LVEF, LV end-diastolic volume index (LVEDVi), LV mass index, left atrial volume index, and we estimated pulmonary artery systolic pressure, early diastolic mean velocity of the mitral annulus (mean e′), and the ratio of the early diastolic velocity of the mitral inflow to e′ (mean E/e′), and inferior vena cava (IVC) diameter. Echocardiography was performed using Vivid 7 (GE Healthcare Japan, Tokyo, Japan) by an experienced, certified ultrasonographer who was blinded to the patient group.

### Statistical Analyses

We performed univariate analysis and subsequent stepwise multivariate analysis with clinical variables of age, laboratory data (hemoglobin, albumin, eGFR, sodium, uric acid, and total cholesterol), comorbidities (hypertension, diabetes mellitus, coronary artery disease, and atrial fibrillation), and medication (ACE inhibitor, angiotensin II type I receptor blocker, mineralocorticoid receptor antagonist, β blocker, diuretics, digitalis, and statins) using GraphPad Prism version 5.02 (Graphpad Software, Inc, La Jolla, CA, USA) and SPSS version 10 (IBM, Armonk, NY, USA). The exploratory factor analysis was performed using JMP 10 (SAS Institute, Cary, NC, USA). The number of principal components was first decided by a scree plot, and the factors were extracted by Quartimin oblique rotation of the principal components.

All results are presented as median and interquartile range unless otherwise stated. Statistical significance was evaluated using the two-tailed Mann–Whitney *U*-test for comparisons between two median values. The correlation coefficient of the variables was calculated as Spearman’s rank correlation coefficient. P-values of < 0.05 were considered statistically significant.

## Results

### Plasma AA profiling

We first investigated changes in the plasma concentration or the amount of 41 AAs and Fischer ratio in the control and HF groups. The HF group (n = 38) was older than the control group (n = 33), and eGFR was significantly lower in the HF group than in the control group (Table 1). In the HF group, the median LVEF was 30.9%, and about two-thirds of these patients had chronic kidney disease and nearly half had coronary artery disease and atrial fibrillation.

**Table 1:** Baseline clinical characteristics in the Control and HF groups.

Clinical Variables	Control (n = 33)	HF (n = 38)	p-value
age (years)	52.0 (44.0, 68.0)	73.0 (60.8, 77.0)	0.0001
sex (male, %)	87.9	76.3	0.22
BMI	22.8 (21.8, 25.1)	22.0 (20.0, 24.4)	0.17
smoking (%)	33.3	23.7	0.42
laboratory data			
eGFR (mL/min/1.73 m^2^) (ml/min/1.73 m^2^)	73.1 (67.2, 76.9)	50.3 (38.2, 63.5)	<0.0001
BNP (pg/mL)	8.3 (4.0, 20.1)	290.5 (183.5, 679.3)	<0.0001
LVEF (%)		30.9 (21.5, 40.5)	
comorbidity (%)			
hypertension	24.2	81.6	<0.0001
diabetes mellitus	3	34.2	0.001
chronic kidney disease	9.1	65.8	<0.0001
coronary artery disease	0	47.4	N/A
atrial fibrillation	3	44.7	<0.0001
medication (%)			
ACEI/ARB/MRA	15.2	76.3	<0.0001
β-blocker	6.1	55.3	<0.0001
diuretics	3	73.7	<0.0001
digitalis	0	26.3	N/A
statin	6.1	31.6	<0.01

Data are median and interquartile range (in parentheses) unless otherwise stated. ACEI, angiotensin-converting enzyme inhibitor; ARB, angiotensin II type I receptor blocker; BMI, body mass index; MRA, mineralocorticoid receptor antagonist; N/A, not applicable

The plasma concentration of AAs was measured by high-performance liquid chromatography in these participants, and we found that 17 of 41 AAs and two ratios significantly changed in the HF group compared with those in the control group (p < 0.05, Table 2). In the HF group, amounts of histidine, tryptophan, and Fischer ratio decreased, whereas those of other factors increased.

**Table 2:** Plasma AAs and ratios—amounts significantly changed in the HF group.

AAs and Ratios	Control	HF	p-value
Hydroxyproline	10.2 (8.9, 12.9)	14.1 (11.1, 16.4)	<0.001
Serine	115.3 (106.7, 121.4)	123.8 (105.7, 140.6)	<0.05
Asparagine	52.7 (47.7, 58.7)	59.6 (53.5, 68.0)	<0.01
Glutamate	58.0 (44.5, 65.2)	79.0 (53.8, 100.4)	<0.001
Glycine	229.7 (202.6, 253.8)	247.5 (215.3, 297.8)	<0.05
Citrulline	35.0 (28.1, 40.3)	47.1(33.6, 52.4)	<0.01
Cystine	52.6 (48.8, 58.5)	68.5 (56.1, 87.0)	<0.0001
Tyrosine	61.5 (55.3, 72.6)	73.4 (59.5, 84.5)	0.01
β-alanine	4.3 (3.3, 5.4)	5.5 (4.2, 8.3)	<0.01
Phenylalanine	60.7 (56.9, 69.8)	71.3 (62.6, 85.3)	<0.01
β-aminoisobutyric acid	2.3 (1.7, 3.5)	4.9 (3.0, 7.5)	<0.001
MEA	9.0 (8.2, 10.4)	10.3 (8.9, 12.3)	<0.05
Ornithine	67.0 (59.2, 71.3)	89.4 (71.6, 107.7)	<0.0001
O:A ratio	0.78 (0.66, 0.94)	1.06 (0.76, 1.16)	<0.001
1-methylhistidine	4.0 (2.4, 5.5)	23.3 (10.1, 33.8)	<0.0001
Histidine	85.8 (80.7, 91.5)	80.1 (66.7, 88.6)	0.01
3-methylhistidine	4.3 (4.0, 5.2)	9.1 (7.7, 15.9)	<0.0001
Tryptophan	53.9 (49.1, 57.9)	46.2 (40.4, 54.8)	<0.01
Fischer ratio	3.3 (3.1, 3.6)	3.1 (2.6, 3.5)	<0.05

Data are median and interquartile range (in parenthesis). Units are μM except Ornithine:Arginine ratio and Fischer ratio. MEA, monoethanolamine; O:A ratio, Ornithine:Arginine ratio

### Specific AAs and Fischer ratio correlated with cardiac function in the HF group

Subsequently, we performed univariate and stepwise multivariate analyses with clinical parameters of age, laboratory data related to the prognosis of HF, comorbidities, and medications to identify correlations of concentrations of AA with cardiac function in the HF group. Univariate analysis revealed that 11 of 17 AAs and two ratios were significantly correlated with LVEF, IVC diameter, mean mitral e′, mean mitral E/e′, and BNP value (p < 0.05, Table 3). We additionally examined the correlation of AAs, the concentration of which was unchanged in the control and HF groups, with cardiac function. As a result, we found that methionine and glutamine were significantly associated with LVEDVi and IVC diameter, respectively (p < 0.05).

**Table 3:** Correlation of specific AAs and ratios with cardiac function in the HF group.

Cardiac Function	AAs and Ratios	Univariate		Stepwise Multivariate		
		Spearman ρ	p-value	Adjusted R-square	β	p-value
LVEF	MEA	−0.47	<0.01	0.58	−0.56	0.001
	Glutamate	−0.43	<0.05			
	Fischer ratio	0.36	<0.05			
	Phenylalanine	−0.34	0.06			
	Tryptophan	−0.34	0.06			
LVEDVi	Methionine	−0.48	<0.01	0.39	−0.44	0.01
IVC diameter	Tyrosine	0.55	<0.01	0.4	0.47	<0.05
	O:A ratio	0.54	<0.01			
	Tryptophan	0.48	<0.01			
	Glutamine	0.46	<0.05			
	MEA	0.41	<0.05			
mean mitral e′	Fischer ratio	−0.45	<0.05			
	β-alanine	−0.39	0.07			
mean mitral E/e′	3-methylhistidine	0.61	<0.01			
	1- methylhistidine	0.49	<0.05	0.53	0.49	<0.01
	β-alanine	0.4	0.07			
BNP	Histidine	−0.47	<0.01	0.3	−0.34	<0.05
	Fisher ratio	−0.37	<0.05	0.3	−0.33	<0.05

β, standardized regression coefficient; ρ, correlation coefficient

**Fig 1:**
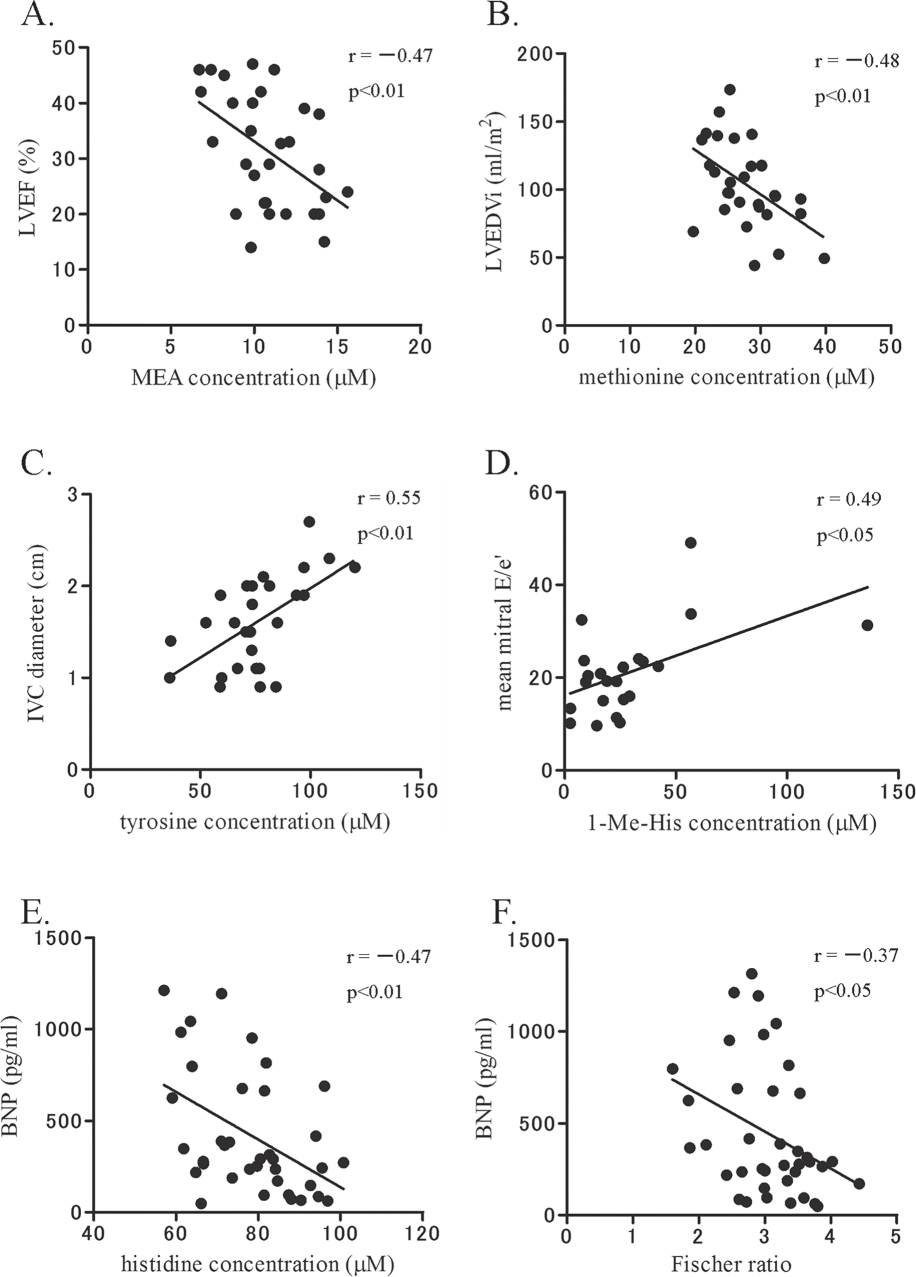
Specific AAs and Fischer ratio were significantly correlated with cardiac function in the HF group. Of 41 AAs examined, the amounts of five AAs and Fischer ratio were significantly correlated with cardiac function by univariate and stepwise multivariate analyses (p < 0.05). Their amounts, except that of methionine, significantly changed between the control and HF groups. r, correlation coefficient.

We performed stepwise multivariate analysis of these cardiac function-related AAs and ratios with clinical variables and found that five specific AAs [ie, monoethanolamine (MEA), methionine, tyrosine, 1-methylhistidine (1-Me-His), and histidine] and Fischer ratio significantly correlated with cardiac function (p < 0.05, Table 3 and Fig. 1). In the subjects ≧60 years old, whose age ranges for the control and HF groups were not significantly different (p = 0.10), correlation of AAs with cardiac function revealed a similar profile, although only ornithine to arginine ratio correlated with IVC diameter by multivariate analysis (β = 0.62, p = 0.001, data not shown). Moreover, we found that in ischemic HF (n = 18) Fischer ratio was significantly correlated with LVEF and mean mitral e′by multivariate analysis (β = 0.61 and −0.68, p < 0.01 and 0.001, respectively, data not shown).

### Exploratory factor analysis categorized cardiac function-related AAs by two components in the HF group

**Fig 2:**
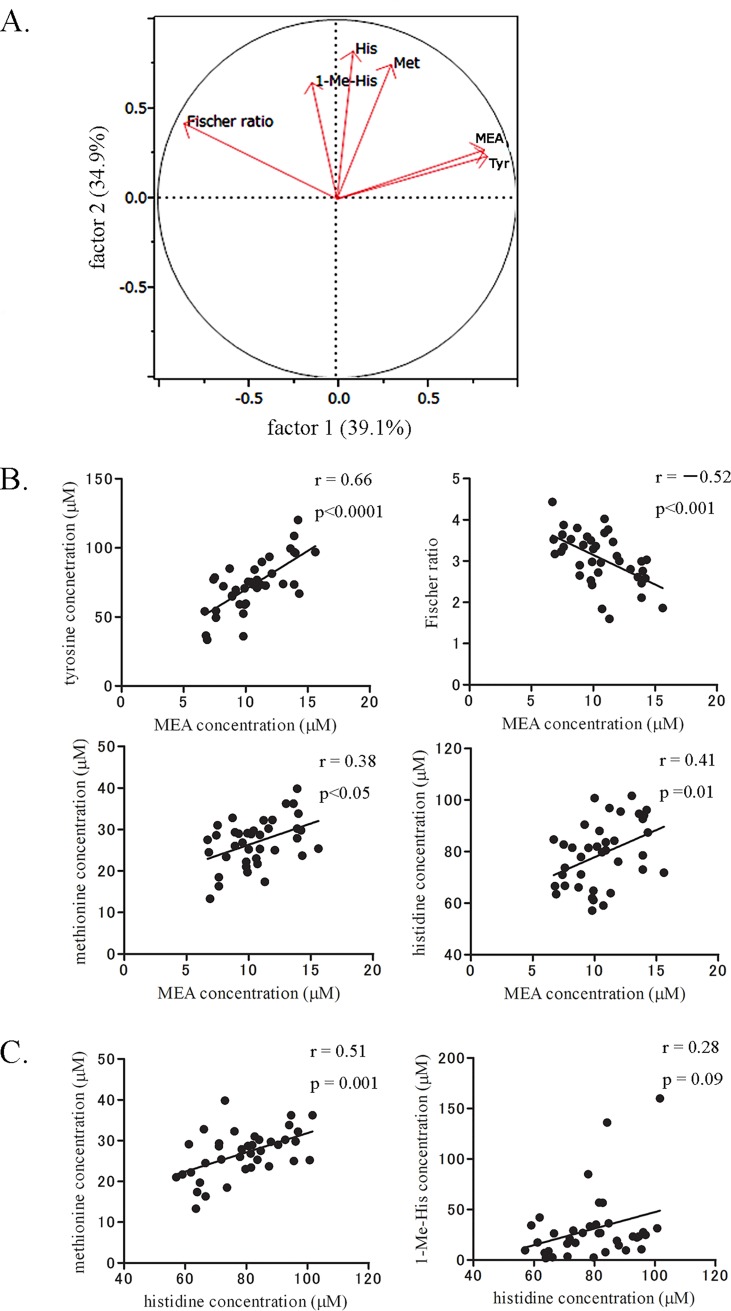
Exploratory factor analysis categorized cardiac function-related AAs by two components in the HF group. A. A two-dimensional plot of factor loading. Cardiac function-related AAs and Fischer ratio in Fig. 1 were subjected to exploratory factor analysis that identified two potential factors by which AAs and ratio could be categorized: factor 1 (MEA, tyrosine, and Fischer ratio) and factor 2 (histidine, methionine, and 1-Me-His). The percentages in parentheses and values of the axes denote a contribution ratio of the factors and a factor loading, respectively. B and C. The correlations between AAs and Fischer ratio in A. Correlations of MEA with methionine and histidine and that of histidine and 1-Me-His were relatively weak.

To explore potential factors to categorize these five specific AAs and Fischer ratio, we conducted a factor analysis. The analysis indicated that two potential factors (factor 1 and factor 2) can reasonably categorize these AAs and Fischer ratio with a contribution ratio of 74% (Fig. 2A). MEA categorized to factor 1 was significantly correlated with tyrosine (r = 0.66, p < 0.0001) and Fischer ratio (r = −0.52, p < 0.001), but it showed relatively weak or no correlation with AAs in factor 2, ie, methionine (r = 0.38, p < 0.05) and histidine (r = 0.41, p = 0.01) (Fig. 2B) or 1-Me-His (r = 0.08, p = 0.65). In contrast, histidine was significantly correlated with methionine (r = 0.51, p = 0.001) and showed weak correlation with 1-Me-His (r = 0.28, p = 0.09) (Fig. 2C).

Interestingly, the categorized AAs and Fischer ratio were specifically correlated with hepatic function, BCAAs that indicate skeletal muscle metabolism, and renal function. MEA, tyrosine, and Fischer ratio in factor 1 were significantly correlated with hepatic function as indicated by total bilirubin, aspartate aminotransferase, alanine aminotransferase, and MELD-XI score (p < 0.01, Table 4). In contrast, histidine and methionine in factor 2 were modestly associated with hepatic function but strongly correlated with BCAAs (r = 0.60–0.72, p < 0.0001; data not shown). 1-Me-His in factor 2 was moderately correlated with leucine (r = 0.39, p < 0.05), isoleucine (r = 0.42, p = 0.01), and renal function of cystatin C and eGFR (Table 4).

**Table 4:** Correlation of cardiac function-related AAs with hepatorenal function in the factor analysis.

AAs and Ratio	Hepatorenal Function	Spearman ρ	p-value
MEA	TB	0.55	<0.001
	MELD-XI (eGFR ≥ 40)	0.52	<0.01
	AST	0.45	<0.01
	ALT	0.4	0.01
Tyrosine	TB	0.56	<0.001
	AST	0.45	<0.01
	ALT	0.4	0.01
	cystatin C	−0.43	<0.01
Fischer ratio	AST	−0.67	<0.0001
	TB	−0.58	0.0001
	ALT	−0.49	<0.01
Histidine	MELD-XI (eGFR ≥ 40)	0.47	<0.05
Methionine	MELD-XI (eGFR ≥ 40)	0.39	<0.05
	AST	0.33	<0.05
1-methylhistidine	cystatin C	0.52	0.001
	eGFR	−0.46	<0.01

TB, total bilirubin; AST, aspartate aminotransferase; ALT, alanine aminotransferase

In skeletal muscle, histidine is metabolized to 1-Me-His and 3-Me-His and is released outside the cell following injury or muscle catabolism [29]. Therefore, these findings suggest that factor 1 and factor 2 are markers of hepatic function and skeletal muscle metabolism, respectively, highlighting the cardio-hepatic-skeletal muscle axis in HF.

### Cardiac function-related AAs revealed unique correlations with concomitant systemic factors in the HF group

Next, we investigated the correlation of the categorized AAs and Fischer ratio with concomitantly identified systemic factors such as oxidative stress, serum NO concentration, inflammation, endothelial function, and nutritional status that could be modifiers or prognostic markers in HF. Serum NO concentration strikingly decreased in the HF group compared with that in the control group (p < 0.0001), whereas serum levels of hydroperoxide as an oxidative stress indicator significantly increased (p < 0.0001), leading to a marked decrease in the serum NO:hydroperoxide ratio (p < 0.0001) (Table 5). Furthermore, flow-mediated dilatation was less than the normal level, and indexes of nutritional status such as serum albumin concentration and GNRI decreased in the HF group (p < 0.001).

**Table 5:** Measured values of concomitant systemic factors in the control and HF groups.

Parameters	Control	HF	p-value
serum NO_2_^−^ + NO_3_^−^ (μM)	13.0 (10.1, 20.2)	4.3 (2.6, 7.4)	<0.0001
Serum hydroperoxide (U.CARR)	283.0 (223.0, 349.0)	421.0 (349.5, 519.0)	<0.0001
serum NO_2_^−^ + NO_3_^−^:hydroperoxide ratio	5.2 (3.0, 7.0)	1.1 (0.6, 1.7)	<0.0001
serum hsCRP (mg/dL)	0.04 (0.02, 0.12)	0.28 (0.11, 0.50)	<0.0001
%FMD		3.5 (2.6, 5.2)	N/A
baPWV (cm/s)		1573 (1419, 1850)	N/A
serum albumin (mg/dL)	4.4 (4.2, 4.6)	3.8 (3.4, 4.2)	<0.0001
GNRI	109.8 (104.7, 114.8)	100.1 (91.0, 107.7)	<0.001

Data are median and interquartile range (in parentheses). baPWV, brachial-ankle pulse wave velocity; %FMD, flow-mediated dilatation

**Fig 3:**
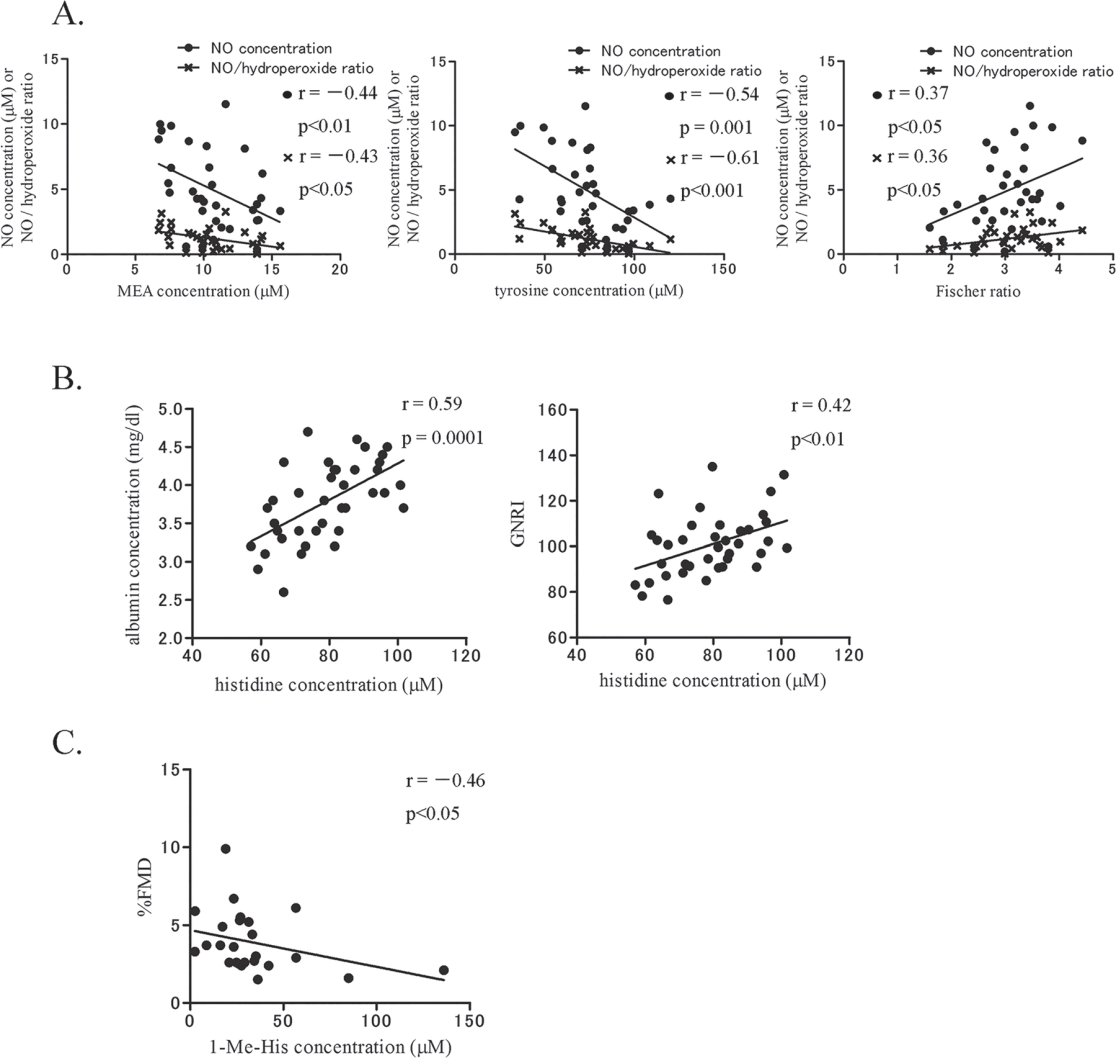
Cardiac function-related AAs revealed unique correlations with concomitant systemic factors in the HF group. A. Concentrations of hepatic-related AAs in Fig. 2A were significantly associated with serum NO concentration and the NO:hydroperoxide ratio (p < 0.05). B. Concentration of histidine, hepatic- and skeletal muscle-related AA in Fig. 2A, was associated with serum albumin concentration and GNRI (p < 0.01). C. Concentration of 1-Me-His, skeletal muscle- and renal-related AA in Fig. 2A, was negatively associated with flow-mediated dilatation (p < 0.05). %FMD, flow-mediated dilatation.

Surprisingly, the categorized cardiac function-related AAs and Fischer ratio showed unique correlations with systemic factors. MEA, tyrosine, and Fischer ratio as hepatic-related AAs showed not a strong, but significant correlation with serum NO concentration and the NO:hydroperoxide ratio (p < 0.05, Fig. 3A). In the subgroup of ischemic HF, Fischer ratio revealed a trend of relationship with serum NO concentration (ρ = 0.46, p = 0.08, data not shown). Histidine as a hepatic- and skeletal muscle-related AA was significantly correlated with serum albumin concentration and GNRI (p < 0.01, Fig. 3B). In contrast, 1-Me-His as a skeletal muscle- and renal-related AA was negatively correlated with flow-mediated dilatation (p < 0.05, Fig. 3C). Nevertheless, correlations of AAs and Fischer ratio with these factors would be reasonable considering the nature of the categories.

## Discussion

**Fig 4:**
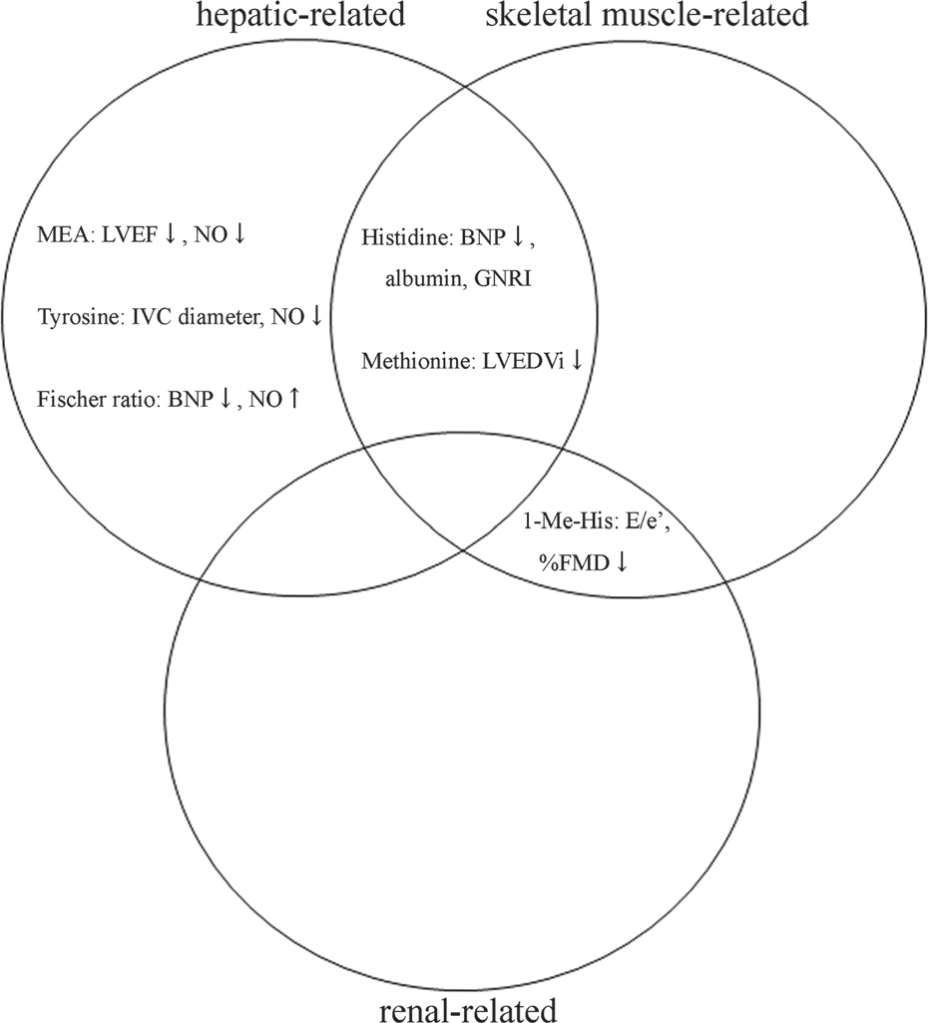
Summary of cardiac function-related AAs on the cardio-hepatic-skeletal muscle axis in patients with systolic HF. Plasma AA profiling revealed that five AAs and Fischer ratio specifically correlated with cardiac function in patients with systolic HF. These AAs can be categorized based on the cardio-hepatic-skeletal muscle axis and had specific correlations with serum NO and albumin concentrations, GNRI, and %FMD.

In the present study, plasma AA profiling highlighted the cardio-hepatic-skeletal muscle axis and identified five specific AAs and Fischer ratio that are significantly correlated with cardiac function of LVEF, LVEDVi, mitral E/e′, IVC diameter, and BNP in patients with systolic HF. These AAs and Fischer ratio showed unique correlations with concomitant systemic modifiers and prognostic markers of HF, such as serum NO concentration, endothelial function, and nutritional status (Fig. 4).

First, we found that nearly half of concentration of AAs significantly changed in the HF group compared with those in the control group. However, univariate and stepwise multivariate analysis with clinically potential confounders identified five AAs and Fischer ratio that are correlated with cardiac function. Subsequent exploratory factor analysis successfully disclosed two potential factors to categorize these cardiac function-related AAs and Fischer ratio. Unexpectedly, these factors were presumed to be hepatic function and skeletal muscle metabolism according to significant correlations with well-known hepatic function tests, MELD-XI score, and BCAAs that reflect skeletal muscle metabolism. Importantly, the categorized AAs and Fischer ratio revealed unique associations with specific modifiers and prognostic markers in HF.

Recently, plasma metabolite profiling using high-resolution mass spectroscopy and nuclear magnetic resonance spectroscopy has facilitated new research in cardiovascular diseases. Researchers have found that plasma AA profiling can predict cardiometabolic risk. Wang et al [21, 22] showed that glutamine, glutamate, glutamine:glutamate ratio, and 5 BCAAs and aromatic AAs (ie, isoleucine, phenylalanine, tyrosine, leucine, and valine) are highly associated with future diabetes in a healthy population. Shah et al [24] further reported that urea cycle-related AAs (i.e., arginine, citrulline, and histidine) and BCAA-related AAs are correlated with coronary artery disease and subsequent cardiovascular events such as death or myocardial infarction. In contrast, AA profiling in HF has been scarcely studied to date, although researchers have reported that the concentration of 5 AAs is decreased and that urinary taurine is associated with worsening renal function [26]. Histidine concentration was decreased with HF, consistent with the current study; however, tyrosine was also decreased in their study, in contrast with the finding in the current study. In Lin’s study, blood samples were examined in patients with end-stage HF within 2 weeks just before heart transplantation. Their average EF (20%) was lower than that in our study, and nearly half of them suffered from liver cirrhosis partly due to HF, indicating disease severity would be higher than that in our study. Moreover, comorbidity rate of diabetes was lower in their study. Therefore, the differences in HF severity and comorbidity possibly reason the discrepancy between the two studies.

The findings in the present study support several hypotheses in systolic HF. First, we noticed not a strong, but significant correlation of hepatic function-related AAs with serum NO concentration. This finding may implicate NO regulation of hepatic function possibly by reducing congestion or by activating downstream endothelial NO synthase/Akt signaling in the liver, or hepatic regulation of NO production in HF [30, 31]. Second, we found that histidine and Fischer ratio were significantly decreased and were associated with BNP values in HF. Although this finding may be just an epiphenomenon, one preclinical study in rodents has demonstrated that supplementation with BCAAs potentially could improve cardiac function [32]. Considering the result of ischemic subgroup analysis in this study, these hypotheses could be mainly applied to ischemic HF. Third, 1-Me-His was correlated with skeletal muscle metabolism and renovascular function. This finding may help to assess the risk or therapeutic efficacy of treatments for skeletal muscle wasting and endothelial dysfunction. Therefore, our study emphasizes plasma AA profiling as a possible useful tool to explore markers of risk assessment and prognosis, mechanistic insights, and therapeutic targets in HF. However, in nature of the single-center, case-control study, additional questions would need to be answered by larger studies before the identified AAs could be used for clinical decision-making, such as whether these AAs change with disease severity, and does AA-directed therapy improve outcomes.

In this study, skeletal muscle volume and function were not directly investigated by methods such as circumferential length of the brachial muscle, dual-energy X-ray absorptiometry, bioelectrical impedance, cardiopulmonary exercise test, and 6-min walking distance. The prescription rates of ACE inhibitors, angiotensin II type I receptor blockers, or β-blockers to the HF patients seemed less than expected. However, this can be explained by the fact that the study participants had not received initial treatment for HF.

In conclusion, the present study demonstrates that plasma AA profiling identifies correlations of specific AAs with cardiac function and concomitant systemic factors, highlighting the cardio-hepatic-skeletal muscle axis in patients with systolic HF. The findings in this study may help researchers to explore novel biomarkers, pathogenesis, and therapeutic targets for HF.
